# Focal Polycomb-Mediated Repression of Neuronal Identity and Synaptic Maintenance Genes in Aging Neurons

**DOI:** 10.7759/cureus.111824

**Published:** 2026-06-30

**Authors:** Ngo Cheung

**Affiliations:** 1 Psychiatry, Cheung Ngo Medical Limited, Hong Kong, HKG

**Keywords:** activity-by-contact, cask, cognitive aging, epigenetic biomarker, h3k27me3, neuroligin, neuronal aging, polycomb, protocadherins, synaptic maintenance

## Abstract

Background: Neuronal aging is accompanied by changes in chromatin regulation, but the gene-level mechanisms that connect epigenetic remodeling to circuit decline remain incompletely defined.

Methods: We performed a secondary, Activity-by-Contact (ABC)-inspired analysis of histone H3 lysine 27 trimethylation (H3K27me3) signal tracks from purified mouse forebrain neurons in GSE190102, focusing on the contrast between young adult neurons at 3 months and old neurons at 24 months, with 12-month samples used to evaluate age trajectories.

Results: We identified 1,500 age-associated region-gene assignments, with a strong predominance of H3K27me3 gain. In total, 1,383 assignments, representing 92% of the interpreted set, showed gain, whereas 117, representing 8%, showed loss. The strongest gain module involved clustered protocadherin genes, including multiple Pcdhb genes, Pcdhgb4, Pcdhgc3, and Pcdha7. A second gain module involved synaptic maintenance and neurotransmission genes, including Nlgn3, Cask, Dlg4, Dlg3, Syn1, Chrm2, Chrna5, and Chrnb4. These changes were accompanied by a smaller loss module involving chromatin, transcriptional, lysosomal, and stress-response candidates, including Med14, Med12, Atp6ap2, and Heph. Functional enrichment highlighted calcium-dependent cell-cell adhesion, cell-adhesion molecules, neuroactive ligand-receptor interaction, and cholinergic synapse pathways.

Conclusions: These results support a working model in which aging neurons undergo Polycomb redistribution rather than simple uniform loss of H3K27me3. In this model, focal hyper-repression of neuronal identity and synaptic scaffold programs may reduce circuit precision, while selected loss of repression at stress and transcriptional regulators may permit compensatory or maladaptive rewiring. We propose a compact candidate 14-gene Synaptic Epigenetic Aging Signature as a practical biomarker and screening panel for cognitive aging, neuromodulatory treatment stratification, and future locus-specific epigenome-editing studies.

## Introduction

The challenge of early synaptic decline in brain aging

Cognitive aging does not begin only when neurons die. A substantial body of neuropathological and experimental work indicates that synaptic deterioration, dendritic spine remodeling, and network-level instability can emerge before overt neurodegeneration. In Alzheimer’s disease and related states, synapse loss has repeatedly shown a close relationship with cognitive impairment, sometimes more direct than classical lesion burden alone [1,2]. Work on the aging cortical synapse has also emphasized that age-related cognitive decline is often best understood as a failure of synaptic maintenance, plasticity, and circuit tuning rather than a simple loss of cell number [3]. Dendritic spine changes during normal aging further support the view that structural weakening of neuronal connectivity is a central event in age-related functional decline [4].

This creates a practical problem for early detection. Common clinical and translational biomarkers, including amyloid and tau measures, neurofilament light, inflammatory markers, and structural imaging, are useful, but they often index established Alzheimer’s disease biology, neurodegeneration, inflammatory state, or downstream neuronal injury rather than the earliest loss of synaptic specificity [5-7]. They may not fully capture the earlier loss of neuronal individuality, synaptic specificity, and adaptive responsiveness that precedes more obvious degeneration [1-4]. A molecular readout that reports on synaptic vulnerability before large-scale synapse loss would therefore have high value.

Epigenetic mechanisms are attractive candidates for such a readout because they sit between long-lived cellular identity and environmental or activity-dependent change. Among these mechanisms, Polycomb regulation and H3K27me3 are especially relevant. Polycomb repressive complex 2 (PRC2) catalyzes methylation of histone H3 at lysine 27, and Polycomb systems help maintain stable transcriptional states while allowing controlled developmental transitions [8,9]. In aging neurons, however, the field has largely focused on broad loss of chromatin marks or global reconfiguration. The possibility that aging involves a more selective redistribution of repression, with focal gain at genes needed for synaptic precision, has received less attention.

Clustered protocadherins as molecular barcodes for neuronal identity

Clustered protocadherins are among the most compelling gene systems for linking chromatin regulation to neuronal identity. The alpha, beta, and gamma protocadherin clusters encode cell-surface molecules that can be expressed in combinatorial patterns across individual neurons. These combinations create a molecular recognition system that helps neurons distinguish self from non-self, space their dendrites, and refine local circuits. Protocadherins are therefore often described as part of a neuronal barcode system, although this shorthand should not obscure the complexity of the underlying biology [10,11].

Experimental studies have shown that protocadherins are required for dendritic self-avoidance in the mammalian nervous system, a process by which branches from the same neuron repel one another and distribute across space [12]. The combinatorial interaction of alpha, beta, and gamma protocadherins can generate single-cell identity through homophilic binding, giving neurons a way to maintain spatial and synaptic specificity [11]. Developmental and epigenetic regulation of clustered protocadherin expression contributes to stochastic isoform choice and neuronal diversity [13]. CCCTC-binding factor (CTCF)-dependent chromatin architecture is also required for proper clustered Pcdh expression in neurons, linking three-dimensional genome organization to the generation of neuronal individuality [14].

These features make the clustered protocadherin loci unusually sensitive indicators of circuit identity. If aging neurons gain repressive H3K27me3 at selected protocadherin genes, the predicted consequence is not simply lower expression of one adhesion molecule. It may be a compression of the combinatorial surface code that helps neurons maintain self/non-self recognition and synaptic partner discrimination [8-14].

Synaptic adhesion and scaffold machinery in aging

Protocadherins define one layer of neuronal identity, but mature synapses also depend on adhesion molecules, scaffold proteins, vesicle regulators, and neurotransmitter receptors. Neuroligin-3, encoded by Nlgn3, participates in trans-synaptic adhesion through neurexin-neuroligin complexes. These complexes help organize synapse specification, synaptic function, and circuit logic [15]. CASK is another important synaptic organizer. It is a membrane-associated guanylate kinase protein that interacts with neurexins and participates in synaptic protein complexes that connect adhesion to vesicle release machinery [16,17].

Postsynaptic density proteins also provide a structural foundation for synaptic stability. PSD-95, encoded by Dlg4, and PSD-93, encoded by Dlg3, belong to the membrane-associated guanylate kinase (MAGUK) family and help organize glutamate receptor anchoring, synaptic architecture, and postsynaptic signaling [18,19]. Synapsin-1, encoded by Syn1, regulates synaptic vesicle pools and contributes to neurotransmitter release and short-term plasticity [20]. Together, these genes form a practical core of synaptic maintenance machinery.

Aging also affects neuromodulatory tone. The cholinergic hypothesis of geriatric memory dysfunction proposed that a decline in cholinergic signaling contributes to age-related memory deficits [21]. Later work has refined this view, showing that cholinergic systems are involved in attention, plasticity, and neurodegenerative vulnerability [22]. Nicotinic and muscarinic receptor changes are therefore not isolated receptor events; they may shape whether an aging circuit remains responsive to cognitive demand and pharmacological intervention.

Polycomb/H3K27me3 in neuronal aging and the redistribution hypothesis

Polycomb repression is classically linked to developmental gene control. In embryonic stem cells, Polycomb complexes directly repress large sets of developmental regulators, many of which encode transcription factors that would otherwise promote differentiation [23]. PRC2 deposits H3K27me3, while PRC1 contributes to compaction and H2A ubiquitylation; together, these systems help stabilize cell identity while preserving controlled plasticity [9,24].

The GSE190102 source study mapped H3K4me3, H3K27ac, and H3K27me3 in purified mouse neurons across adulthood and old age. The authors reported stable H3K4me3, global losses of H3K27ac and H3K27me3 into old age, loss of H3K27ac at synaptic signaling genes, and loss of H3K27me3 at developmental gene regulatory regions [25]. These findings suggest broad chromatin reconfiguration in aged neurons. They do not, however, exclude the possibility that focal gains occur at selected loci. In fact, environmentally induced memory impairment has previously been associated with genome-wide redistribution of H3K27me3, showing that this mark can be remodeled in neuronal contexts rather than simply lost uniformly [26].

We therefore tested the hypothesis that aging neurons undergo Polycomb redistribution: focal gain of H3K27me3 at connectivity and synaptic identity genes, alongside selective loss at developmental, stress-response, or transcriptional regulator loci.

Translational gap and study rationale

Building on work in synaptic aging, protocadherin-mediated neuronal identity, Polycomb biology, and the GSE190102 neuronal chromatin dataset, there is currently no compact framework that connects locus-specific H3K27me3 remodeling to early synaptic aging, biomarker development, drug screening, and treatment stratification [1-4,8-14,25,26]. The present manuscript develops such a framework from a secondary analysis of GSE190102. The central proposal is that focal H3K27me3 gain at protocadherin and synaptic maintenance genes may define a candidate Synaptic Epigenetic Aging Signature for future translational development.

The present study had three primary objectives: (1) to perform a secondary, Activity-by-Contact (ABC)-inspired analysis of H3K27me3 signal tracks from purified mouse forebrain neurons in GSE190102, contrasting 3-month and 24-month samples while using 12-month samples to assess age trajectories [25,27]; (2) to test the hypothesis that neuronal aging involves Polycomb redistribution, with focal gain of repressive H3K27me3 at genes supporting neuronal identity and synaptic maintenance accompanied by selective loss at selected transcriptional and stress-response loci, rather than uniform global loss; and (3) to derive a compact, translationally actionable 14-gene Synaptic Epigenetic Aging Signature suitable for future biomarker development, drug-screening endpoints, and locus-specific epigenome-editing studies.

## Materials and methods

Data source and preprocessing

Processed H3K27me3 signal files were obtained from GSE190102, a Gene Expression Omnibus series containing genome binding and occupancy profiling data from purified mouse forebrain neuronal nuclei. The source study used fluorescence-activated nuclei sorting to isolate NeuN-positive neuronal nuclei from male mouse forebrain and profiled H3K4me3, H3K27ac, and H3K27me3 across adult age points [25,27]. The present analysis focused on H3K27me3 data from 3-, 12-, and 24-month neurons, using the 3-month and 24-month samples as the main young-versus-old contrast and the 12-month samples to evaluate trajectories.

**Figure 1:**
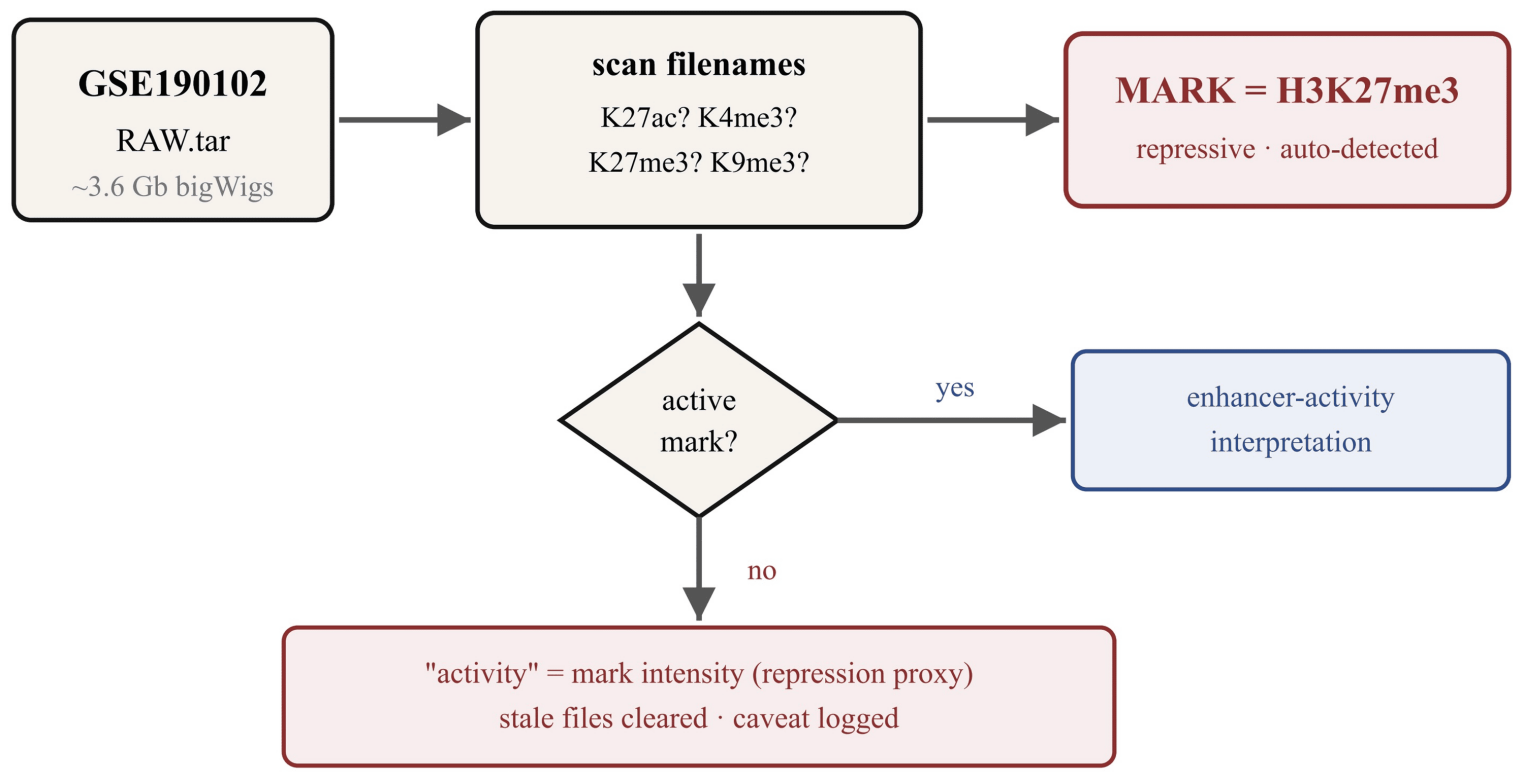
Data ingestion and automatic mark detection The GSE190102 raw archive is retrieved, its bigWig entries are scanned for histone-mark tokens, and the dominant mark is selected; a repressive-mark caveat is raised when no active mark is present. GEO: Gene Expression Omnibus, H3K27me3: histone H3 lysine 27 trimethylation Credits to Ngo Cheung Created using PowerPoint; no AI used

The pipeline (Figure 1) automatically detected the histone mark present in the available signal archive and extracted the H3K27me3 files after clearing stale or unrelated files. Quality control included inspection of chromosome coverage, signal distribution, and sample age labels. A consensus region set was generated by identifying the top 2% signal bins across all samples using 1 kb bins, merging overlapping or nearby intervals with a 500 bp slack, and retaining merged regions of at least 500 bp. This yielded the region universe used for downstream differential, trajectory, and gene-assignment analyses.

Differential and trajectory analysis

**Figure 2:**
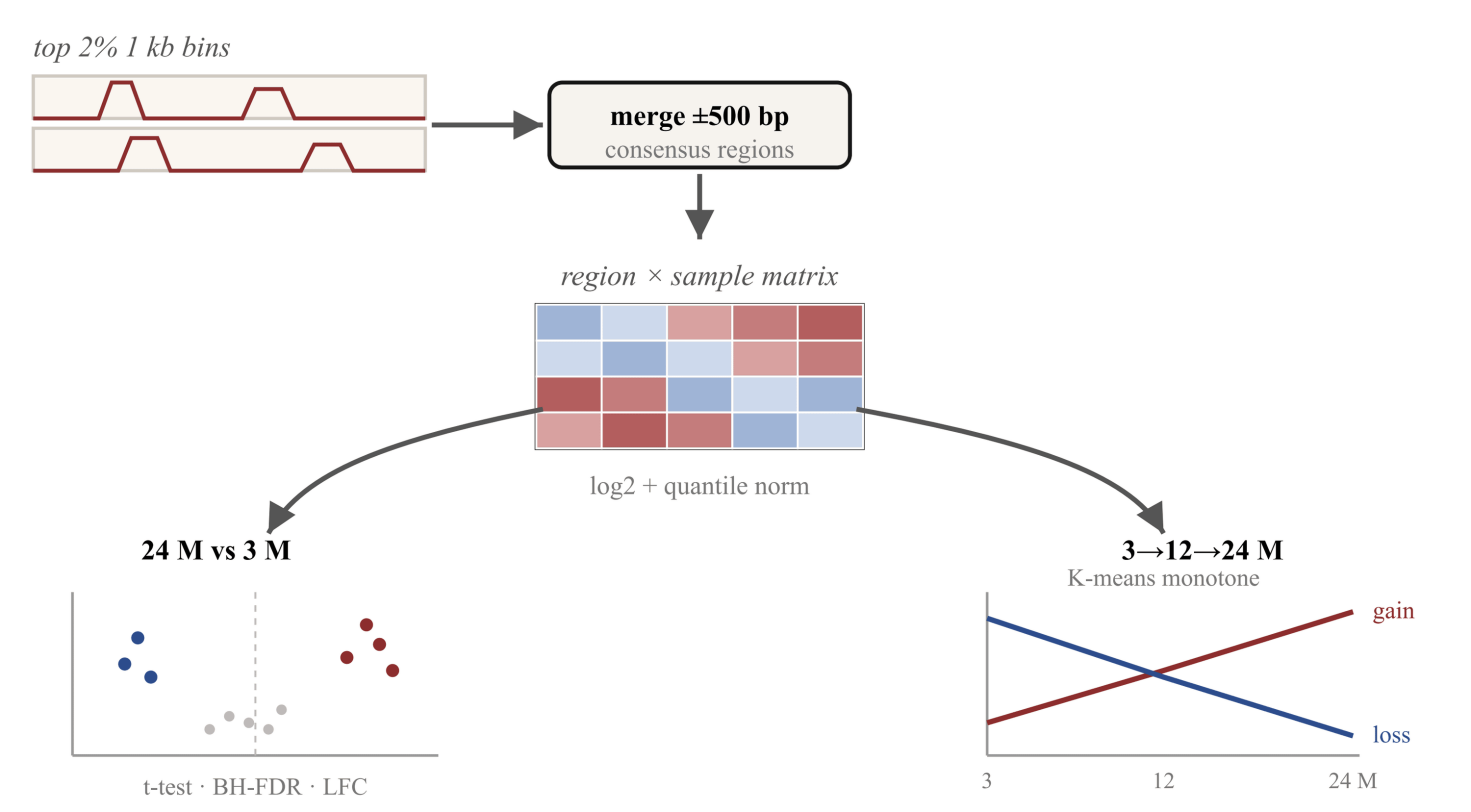
Consensus regions, quantification, and differential/trajectory modeling Top-signal 1 kb bins are merged into a consensus set; signal is quantified per sample, log-transformed and quantile-normalized, and then tested between 24-month and 3-month neurons and clustered across 3, 12, and 24 months. H3K27me3: histone H3 lysine 27 trimethylation, kb: kilobase, M: months Credits to Ngo Cheung Created using PowerPoint; no AI used

H3K27me3 signal was quantified for each consensus region in every sample. The resulting matrix was log2-transformed and quantile-normalized to reduce differences in global signal distribution between samples (Figure 2). Differential analysis between 24-month and 3-month neurons was performed using two-sided t-tests, followed by Benjamini-Hochberg correction for multiple testing [28]. A strict threshold of false discovery rate (FDR) below 0.05 with an absolute log2 fold-change above 0.25 produced too few regions for stable biological interpretation. For this reason, the analysis used a relaxed discovery threshold of nominal P below 0.01 and absolute log2 fold-change above 0.25, followed by a top-N fallback of 2,000 regions when necessary. This makes the analysis best interpreted as a hypothesis-generating, region-prioritization framework rather than a definitive differential binding study. The primary biological analysis reported below used this t-test-based prioritization pipeline.

Progressive age trajectories were evaluated across 3, 12, and 24 months. Mean signal at each age was z-scored, and K-means clustering was performed across a range of k values from 2 to 6. Clusters were then filtered for monotone or near-monotone gain and loss patterns. Regions satisfying these trajectory filters were carried forward for contact-weighted gene assignment.

Activity-by-Contact scoring and gene assignment

**Figure 3:**
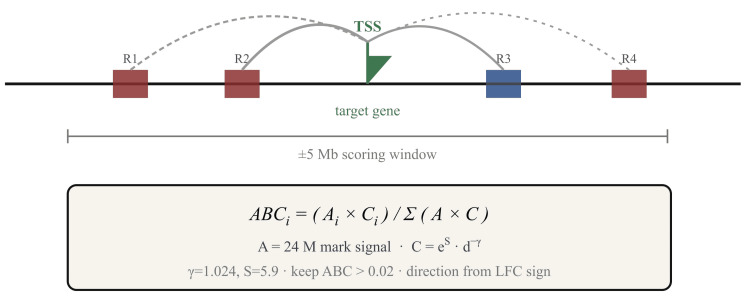
ABC-inspired region-gene assignment For each protein-coding transcription start site from GENCODE vM25, region “activity” based on 24-month H3K27me3 signal is multiplied by a power-law contact term within a 5 Mb window; normalized ABC scores above threshold yield direction-aware region-gene links. ABC: Activity-by-Contact, H3K27me3: histone H3 lysine 27 trimethylation, Mb: megabase, M: months, TSS: transcription start site Credits to Ngo Cheung Created using PowerPoint; no AI used

Gene assignment was performed using an Activity-by-Contact (ABC)-inspired approach (Figure 3) based on the general ABC framework for enhancer-promoter mapping [29]. Because canonical ABC models are usually applied to enhancer activity, the term “activity” is used here in a modified sense: H3K27me3 signal was treated as repressive chromatin activity rather than activating enhancer signal. Contact probability was estimated using a power-law model within a 5 Mb window, with gamma set to 1.024 and scale set to 5.9. The resulting score therefore represented a contact-weighted estimate of which transcription start site (TSS) a repressive H3K27me3 region was most likely to influence.

Protein-coding transcription start sites were derived from GENCODE vM25. Predictions with ABC scores above 0.02 were retained for the main analysis, and sensitivity analyses considered more stringent thresholds up to 0.05. Direction was assigned from the sign of the 24-month versus 3-month log2 fold-change at the associated H3K27me3 region. Positive values were interpreted as H3K27me3 gain, and negative values were interpreted as H3K27me3 loss.

Functional enrichment and network analysis

**Figure 4:**
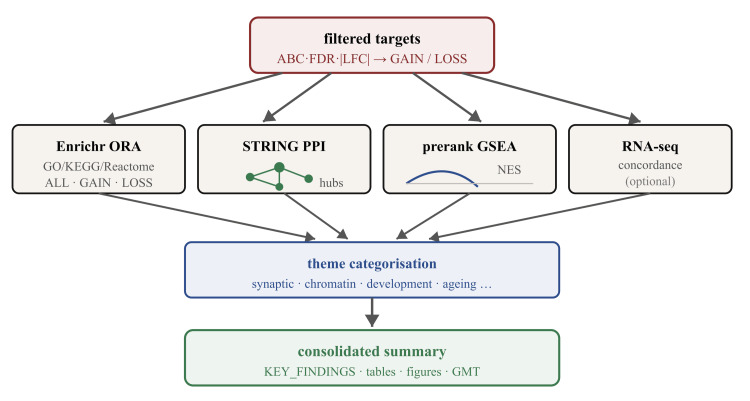
Downstream functional interpretation Filtered, direction-split target genes feed parallel modules: Enrichr over-representation analysis for all targets, gain targets, and loss targets; STRING protein-protein interaction hub detection; preranked gene set enrichment analysis; theme categorization; and optional RNA sequencing concordance, consolidated into an auto-summary. GAIN: H3K27me3 gain, GSEA: gene set enrichment analysis, H3K27me3: histone H3 lysine 27 trimethylation, LOSS: H3K27me3 loss, ORA: over-representation analysis, PPI: protein-protein interaction, RNA-seq: ribonucleic acid sequencing, STRING: Search Tool for the Retrieval of Interacting Genes/Proteins Credits to Ngo Cheung Created using PowerPoint; no AI used

Functional enrichment was conducted separately for all assigned genes, gain-only genes, and loss-only genes (Figure 4). Gene Ontology (GO) Biological Process, Kyoto Encyclopedia of Genes and Genomes (KEGG) mouse pathways, Reactome pathways, and curated gene-set libraries were queried using g:Profiler and Enrichr [30,31]. Enriched terms were grouped into broad biological themes, including cell adhesion, synaptic function, neurotransmission, chromatin regulation, stress response, lysosomal biology, and metal homeostasis. Preranked gene set enrichment analysis (GSEA) was interpreted using the established GSEA framework [32].

Protein-protein interaction (PPI) analysis was performed using STRING with mouse taxon 10090 and a confidence threshold of 0.7 or higher [33]. Network hubs were interpreted cautiously, because high connectivity can reflect literature bias as well as biological centrality. Functional labels were assigned by combining enrichment results, STRING connectivity, and manual review of gene roles in neuronal biology.

Signature definition and validation strategy

The Synaptic Epigenetic Aging Signature was defined by integrating statistical strength, biological coherence, network position, and translational assay feasibility. The primary 14-gene panel comprised Pcdhb10, Pcdhb14, Pcdhgb4, Pcdhgc3, Nlgn3, Cask, Dlg4, Dlg3, Syn1, Chrm2, Chrna5, Chrnb4, Med14, and Atp6ap2. This panel captures the dominant gain events at protocadherin and synaptic maintenance genes, together with representative loss events at transcriptional and lysosomal regulators. Heph, Med12, Tspan7, and Msn were retained as secondary candidates for expanded panels, especially where iron handling, synaptic membrane remodeling, or cytoskeletal adaptation are of interest.

The proposed validation strategy combines targeted H3K27me3 cleavage under targets and tagmentation (CUT&Tag)-quantitative polymerase chain reaction (qPCR) with matched RNA expression. CUT&Tag is well suited to small samples and single-cell-compatible chromatin profiling [34]. For protocadherin loci, isoform-aware RNA sequencing or long-read RNA sequencing is preferable, because total cluster expression may miss changes in isoform diversity. Validation should prioritize Pcdhb10, Pcdhb14, Pcdhgb4, Pcdhgc3, Nlgn3, Cask, Dlg4, Dlg3, Syn1, Chrm2, Chrna5, Chrnb4, Med14, and Atp6ap2, with Heph added when stress and metal-homeostasis biology are being tested.

Statistical and reproducibility considerations

Multiple-testing correction was applied using the Benjamini-Hochberg method [28]. Sensitivity analyses were performed across ABC-score thresholds, fold-change thresholds, and trajectory filters. Because relaxed differential thresholds and top-N fallback were used to generate a biologically interpretable discovery set, all conclusions are presented as mechanistic hypotheses requiring experimental validation.

The primary reported workflow used log2 transformation, quantile normalization, two-sided t-tests, Benjamini-Hochberg correction, a power-law contact model with gamma = 1.024 and scale = 5.9, a 5 Mb assignment window, ABC threshold 0.02, STRING taxon 10090 with confidence of 0.7 or higher, and ORA/GSEA cutoffs of adjusted P < 0.05 and FDR q < 0.25, respectively. Source identifiers and annotation resources included GSE190102, GENCODE vM25, GO Biological Process 2023, KEGG 2019 Mouse, Reactome 2022, Enrichr, g:Profiler, and STRING [25,27,30,31,33]. Exact scripts, package versions, environment information, processed matrices, consensus region lists, gene-assignment tables, signature files, and ABC parameter files will be deposited in a public GitHub repository upon acceptance to support independent replication.

A separate simulation-based benchmarking analysis was performed as a robustness and reproducibility check of the differential-region workflow. This benchmark used a planted-signal dataset to compare limma empirical Bayes modeling, negative-binomial generalized linear modeling, and Welch testing under known-truth conditions [35-37]. These simulations were used to assess pipeline behavior and threshold sensitivity; they did not replace the t-test-based primary biological analysis described above.

## Results

Global landscape and differential H3K27me3 dynamics

The ABC-inspired pipeline identified 1,500 age-associated region-gene assignments in the 24-month versus 3-month comparison. Of these, 1,383 assignments, or 92%, showed H3K27me3 gain, while 117 assignments, or 8%, showed H3K27me3 loss (Table 1). This asymmetry was striking because the source study emphasized global H3K27me3 loss in old neurons, particularly at developmental gene regulatory regions [25]. The two observations are not necessarily contradictory. The source study evaluated broad genome-wide and differential peak behavior, whereas the present analysis focused on contact-weighted assignment of age-associated focal regions within a prioritized signal set. A global reduction in H3K27me3 can coexist with local gain at selected loci if Polycomb repression is redistributed rather than uniformly erased.

**Table 1:** Analysis configuration and global H3K27me3 directional landscape (GSE190102, 24 M versus 3 M) This table summarizes the main configuration used for the age-associated H3K27me3 region-gene assignment analysis and the global directional balance of gain and loss assignments. ABC: Activity-by-Contact, adj P: adjusted P value, conf: confidence, FDR: false discovery rate, GSEA: gene set enrichment analysis, H3K27me3: histone H3 lysine 27 trimethylation, M: months, ORA: over-representation analysis, STRING: Search Tool for the Retrieval of Interacting Genes/Proteins

Parameter	Value
Histone mark	H3K27me3 (repressive)
Gene source	Filtered; fallback top-N by ABC
Analysis genes	1,500 (all with ABC)
Filter	p < 0.01 + top-N
STRING network	36 nodes, 23 edges (conf ≥ 0.7)
ORA/GSEA cutoff	adj P < 0.05/FDR q < 0.25

This distinction is central to the interpretation. The present results do not overturn the conclusion that aged neurons lose H3K27me3 at many developmental loci. Instead, they suggest that the aged neuronal H3K27me3 landscape may be reorganized. In this reorganization, some regions lose repression, especially developmental or regulatory loci, while other regions gain repression at genes linked to neuronal identity, synaptic recognition, and neurotransmission. Such a pattern is consistent with a working model in which aging may reduce the precision of neuronal identity while opening or destabilizing other transcriptional programs. It also resembles broader concepts of age-associated chromatin reconfiguration, in which regulatory information is not simply lost but redistributed across the genome.

The strongest gain signals were not randomly distributed. They clustered around protocadherin genes and synaptic maintenance genes, two gene groups that are highly relevant to the maintenance of neuronal individuality and circuit function. The smaller loss set was enriched for genes involved in transcriptional regulation, lysosomal function, and stress-linked homeostasis. Together, these results support a dual model of aging-associated H3K27me3 dynamics: focal hyper-repression at connectivity genes and selective loss at regulatory or stress-response loci (Tables 2, 3).

**Table 2:** Top 20 genes by ABC score Genes are ranked by the highest retained ABC-inspired score in the direction-aware H3K27me3 region-gene assignment output. Direction indicates the sign of the 24-month versus 3-month H3K27me3 change at the assigned region. ABC: Activity-by-Contact, Dir.: direction, H3K27me3: histone H3 lysine 27 trimethylation

#	Gene	ABC	Dir.
1	Rpgr	1.0	LOSS
2	Vsig4	1.0	LOSS
3	Msn	1.0	LOSS
4	Atp6ap2	1.0	LOSS
5	Med14	1.0	LOSS
6	Mid1ip1	1.0	LOSS
7	Las1l	1.0	LOSS
8-13	H2al1f/c/d/e/b/a	1.0	LOSS
14	Hsf3	1.0	LOSS
15-18	H2al1j/h/g/i	1.0	LOSS
19	Heph	1.0	LOSS
20	Tspan7	1.0	LOSS

**Table 3:** STRING PPI hubs (by degree) Candidate PPI hubs were identified from the STRING mouse network using a confidence threshold of 0.7 or higher. Degree reflects the number of retained network connections for each gene in this filtered network. ABC: Activity-by-Contact, deg: degree, Dir.: direction, PPI: protein-protein interaction, STRING: Search Tool for the Retrieval of Interacting Genes/Proteins Gene symbols follow standard mouse gene nomenclature.

Gene	deg	ABC	Dir.
Tceanc	3	0.666	GAIN
Ddx3x	2	0.870	GAIN
H2ap	2	0.855	GAIN
Nlgn3	2	0.768	GAIN
Med14	2	1.000	LOSS
Cask	2	0.870	GAIN
H2al1a	2	1.000	LOSS
H2al3	2	0.855	GAIN
H2al1i	2	1.000	LOSS
Med12	1	0.768	GAIN
Ar	1	0.624	LOSS
Ophn1/Eda2r	1	0.62-1.0	LOSS

Module A: Protocadherin hyper-repression

The most coherent and biologically compelling signal was the gain of H3K27me3 at clustered protocadherin genes. Multiple beta-protocadherin genes showed gain, including Pcdhb2, Pcdhb3, Pcdhb4, Pcdhb5, Pcdhb6, Pcdhb9, Pcdhb10, Pcdhb11, Pcdhb13, Pcdhb14, and Pcdhb16. Gain was also observed at gamma-protocadherin genes, including Pcdhgb4 and Pcdhgc3, and at the alpha-protocadherin gene Pcdha7. This pattern is important because it represents a coordinated cluster-level event rather than isolated single-gene noise.

Functional enrichment supported the same conclusion. The top enriched Gene Ontology term was calcium-dependent cell-cell adhesion via plasma membrane cell adhesion molecules, with adjusted P = 1.24 × 10^-4 in the overall analysis and adjusted P = 4.04 × 10^-5 in the gain-only analysis (Tables 4-6). This enrichment was driven largely by clustered protocadherin genes. Because protocadherins depend on calcium-mediated extracellular interactions and participate in homophilic recognition, this enrichment has a direct mechanistic interpretation rather than being a generic annotation result.

**Table 4:** Over-representation analysis: significant terms by set and library Significant enrichment terms are counted separately for all assigned genes, gain-only genes, and loss-only genes. A dash indicates that no separate direction-specific value was retained for that library. ALL: all assigned genes, GAIN: H3K27me3 gain genes, GO: Gene Ontology, H3K27me3: histone H3 lysine 27 trimethylation, KEGG: Kyoto Encyclopedia of Genes and Genomes, LOSS: H3K27me3 loss genes, Mamm.: mammalian, MGI: Mouse Genome Informatics, MSigDB: Molecular Signatures Database

Library	ALL	GAIN	LOSS
GO Biological Process 2023	3	4	0
GO Molecular Function 2023	0	-	-
GO Cellular Component 2023	0	-	-
KEGG 2019 Mouse	0	0	3
Reactome 2022	0	0	0
MSigDB Hallmark 2020	0	-	-
MGI Mamm. Phenotype L4	0	0	0

**Table 5:** Over-representation analysis: significant terms by direction Direction-specific enrichment terms are shown for H3K27me3 gain and loss assignments. The protocadherin-dominated adhesion terms were concentrated in the gain set, while KEGG terms in the loss set were interpreted cautiously because of histone-gene and stress-response contributions. adj P: adjusted P value, adh.: adhesion, CAM: cell adhesion molecule, Ca²⁺: calcium, dep.: dependent, GAIN: H3K27me3 gain, H3K27me3: histone H3 lysine 27 trimethylation, KEGG: Kyoto Encyclopedia of Genes and Genomes, LOSS: H3K27me3 loss, PM: plasma membrane

Set	Term	adj P
GAIN	Ca²⁺-dep. cell-cell adhesion via PM CAM	4.04e-5
GAIN	Anterograde trans-synaptic signaling	3.89e-3
GAIN	Chemical synaptic transmission	1.38e-2
GAIN	Cell-cell adhesion via PM adh. molecules	1.57e-2
LOSS	Systemic lupus erythematosus (KEGG)	6.15e-8
LOSS	Alcoholism (KEGG)	8.31e-8
LOSS	Necroptosis (KEGG)	2.28e-6

**Table 6:** Top enriched GO Biological Process terms (ALL targets) with leading member genes Adhesion signatures are dominated by clustered protocadherins in the gain direction; the synaptic signaling term spans cholinergic, serotonergic, GABAergic, scaffold, and protocadherin genes. adj P: adjusted P value, CAM: cell adhesion molecule, Ca²⁺: calcium, Comb. Score: combined score, GO: Gene Ontology, PM: plasma membrane Gene symbols are listed as enrichment outputs and follow standard nomenclature.

Term	adj P	Comb. score	Member genes
Ca²⁺-dependent cell-cell adhesion via PM CAM	1.24e-4	138.9	PCDHGB4, PCDHGC3, PCDHB14, PCDHB13, PCDHB11, PCDHB10, SELP, PCDHB2, SELL, PCDHB16, PCDHB6, PCDHB5, PCDHB4, PCDHB3, PCDHB9
Anterograde trans-synaptic signaling (GO:0098916)	2.21e-2	27.9	CHRM2, NLGN3, CHRNA3, CHRNA5, HTR2C, GABARAP, RPS6KA3, SDCBP, GLRA2, PTCHD1, RPS6KA2, CHRNE, PENK, SPG11, GABRQ, CHRNB4, MINK1, HTR1A, SYN1, DLG3, DLG4, PLP1, PCDHB2–16
Cell-cell adhesion via PM adhesion molecules (GO:0098742)	3.51e-2	26.2	TENM1, PCDHGB4, CLDN2/7/20/23, TRO, SLITRK2, GPC4, PCDHA7, PCDHGC3, L1CAM, SELE, SELP, SELL, IL1RAPL1, PLXNB3, PECAM1, PCDHB2–16

The gene-level model is straightforward but powerful. Clustered protocadherins help establish single-neuron diversity through combinatorial expression and homophilic interactions [10,11]. In the mammalian nervous system, protocadherins mediate dendritic self-avoidance, allowing neurites from the same neuron to repel one another and occupy distinct space [12]. Developmental regulation of clustered protocadherin expression contributes to single-neuron diversity, and chromatin architecture is required for normal stochastic expression of Pcdh genes [13,14]. These roles make clustered protocadherins unusually well positioned to translate chromatin change into circuit-level vulnerability.

H3K27me3 is usually associated with transcriptional repression, although its relationship with expression can depend on local chromatin context. Gain of H3K27me3 at protocadherin loci is therefore predicted to reduce expression of selected isoforms or restrict their combinatorial diversity. The most important consequence may not be the complete silencing of any one Pcdh gene. Rather, it may be a narrowing of the available molecular barcode space. If fewer protocadherin combinations are expressed, neurons may lose some of the surface-code diversity needed for self/non-self discrimination, dendritic spacing, and precise partner recognition.

This interpretation gives the protocadherin module special importance. Many age-related synaptic phenotypes are described at the level of spine density, synaptic proteins, or network activity. The protocadherin gain module points to an earlier and more identity-centered mechanism: aging neurons may become less molecularly distinct before they become structurally lost. The hypothesized phenotype is progressive reduction in synaptic specificity, greater risk of local miswiring, impaired maintenance of dendritic architecture, and reduced circuit precision. This provides a plausible candidate epigenetic route from focal Polycomb gain to cognitive aging.

The priority validation targets within this module are Pcdhb10, Pcdhb14, Pcdhgb4, and Pcdhgc3. These genes capture beta- and gamma-cluster involvement and are practical candidates for targeted CUT&Tag-qPCR. The key validation experiment is to confirm a higher H3K27me3 signal in 24-month neurons than in 3-month neurons at these loci. The second experiment is to test whether the gain corresponds to reduced transcript abundance or reduced isoform diversity using isoform-specific RNA sequencing, single-neuron transcriptomics, or long-read RNA sequencing.

Module B: Synaptic scaffold and receptor repression

A second major gain module involved genes required for synaptic adhesion, postsynaptic organization, vesicle regulation, and neurotransmitter responsiveness. Nlgn3 emerged as a prominent gain target and STRING network hub. Neuroligin-3 participates in neurexin-neuroligin trans-synaptic adhesion, a system that helps organize synapse properties and circuit logic [15]. H3K27me3 gain at Nlgn3 is therefore predicted to weaken the flexibility or abundance of trans-synaptic adhesion machinery. In an aged circuit, this could reduce the stability of excitatory and inhibitory synaptic contacts and impair postsynaptic specialization.

Cask also showed H3K27me3 gain and was prioritized as a synaptic scaffold hub. CASK was originally identified as a Dlg/PSD-95 homolog interacting with neurexins and containing a calmodulin-dependent kinase-like domain [17]. It also participates in protein complexes with the potential to couple synaptic vesicle exocytosis to cell adhesion [16]. Gain of H3K27me3 at Cask therefore fits the same broader theme as Nlgn3: repression at genes that connect synaptic recognition to synaptic assembly and function.

The postsynaptic density genes Dlg4 and Dlg3 were also included in the gain module. Dlg4 encodes PSD-95, a central postsynaptic scaffold that helps sustain the molecular organization of the postsynaptic density and supports glutamate receptor localization [18,38]. Dlg3 encodes PSD-93, another MAGUK scaffold with overlapping and distinct roles in postsynaptic architecture. MAGUK proteins regulate receptor targeting, synaptic maturation, and signaling compartmentalization [19,39]. H3K27me3 gain at these loci is predicted to reduce scaffold capacity and make aged synapses less able to maintain receptor anchoring and plasticity.

Syn1 completed the core structural module. Synapsin-1 regulates synaptic vesicle availability and contributes to the reserve pool and short-term plasticity [20]. If H3K27me3 gain reduces Syn1 expression or responsiveness, the likely functional effect would be weaker vesicle mobilization and impaired neurotransmitter release during repeated activity. In combination, gain at Nlgn3, Cask, Dlg4, Dlg3, and Syn1 suggests coordinated repression of an adhesion-scaffold-vesicle axis. This is not a random list of synaptic genes; it is a connected module spanning synaptic contact, postsynaptic architecture, and presynaptic release capacity.

A parallel gain pattern was observed at neurotransmitter receptor genes. The receptor axis included Chrm2, Chrna5, Chrnb4, Htr1a, Htr2c, Glra2, and Gabrq. KEGG enrichment highlighted neuroactive ligand-receptor interaction and cholinergic synapse pathways (Tables 7, 8). Chrm2 encodes the muscarinic acetylcholine receptor M2. Chrna5 and Chrnb4 encode nicotinic acetylcholine receptor subunits. These genes are relevant to aging because cholinergic dysfunction has long been associated with memory decline and neurodegenerative vulnerability [21,22]. Nicotinic receptors and broader acetylcholine signaling also shape attention, plasticity, and cortical state regulation [40,41].

**Table 7:** Prerank GSEA - significant gene sets (FDR < 0.25): KEGG 2019 Mouse (13 sets) Selected KEGG gene sets passing the preranked GSEA FDR threshold are shown. Positive and negative normalized enrichment scores indicate opposite ranking directions in the preranked analysis. FDR: false discovery rate, GSEA: gene set enrichment analysis, KEGG: Kyoto Encyclopedia of Genes and Genomes, NES: normalized enrichment score

Set	NES	FDR
Alcoholism	+2.73	5.0e-3
Systemic lupus erythematosus	+2.65	5.0e-3
Necroptosis	+2.60	5.0e-3
Cholinergic synapse	-2.32	2.21e-2
Olfactory transduction	-2.35	4.42e-2
Parathyroid hormone action	-1.92	9.33e-2
Apelin signaling	-1.70	1.09e-1
Oxytocin signaling	-1.64	1.18e-1

**Table 8:** Prerank GSEA - significant gene sets (FDR < 0.25): GO Biological Process 2023 (9 sets) Selected Gene Ontology Biological Process terms passing the preranked GSEA FDR threshold are shown. The calcium-dependent adhesion term is consistent with the protocadherin-dominated gain module. Ca²⁺: calcium, dep.: dependent, FDR: false discovery rate, GO: Gene Ontology, GSEA: gene set enrichment analysis, NES: normalized enrichment score, PM: plasma membrane, Pos. reg.: positive regulation

Set	NES	FDR
Pos. reg. protein metabolic process	+2.19	1.0e-2
Ca²⁺-dep. cell-cell adhesion via PM	-2.24	1.46e-2
Pos. reg. cell adhesion	-1.91	2.91e-2
Pos. reg. cell migration	-1.92	3.72e-2
Pos. reg. cell motility	-1.98	3.89e-2
Protein localization to nucleus	-1.59	1.37e-1
Endomembrane system organization	-1.52	1.39e-1
Intracellular protein transport	-1.36	2.45e-1

The combined repression of synaptic structure genes and receptor genes may help explain a common translational problem: aged circuits can respond poorly to neuromodulatory therapies even when neurotransmitter levels are pharmacologically increased. If postsynaptic scaffolds, adhesion proteins, and receptor subunits are epigenetically constrained, simply raising transmitter availability may not restore the molecular infrastructure needed for plasticity. In this sense, H3K27me3 gain at Chrm2, Chrna5, Chrnb4, Nlgn3, Cask, and Dlg4 may mark a state in which cholinergic receptor programs are epigenetically constrained, potentially contributing to variable response to cholinergic or synaptic-enhancing interventions; this idea requires direct testing in aged neuronal models. This remains a hypothesis, but it is directly testable by combining chromatin profiling, RNA measurement, receptor pharmacology, and electrophysiology in aged neurons.

Module C: Dual dynamics and LOSS genes

Although the loss set was smaller, it was biologically informative. The 117 loss assignments included Med14 and Med12, which encode Mediator complex components. Mediator integrates regulatory information from transcription factors and contributes to enhancer-promoter communication and transcriptional responsiveness [42]. Loss of H3K27me3 at Med14 or Med12 could permit altered transcriptional flexibility in aged neurons. This may be compensatory if it helps neurons respond to stress, but it may also increase transcriptional noise or shift activity-dependent gene regulation away from youthful patterns.

Atp6ap2 was another notable loss candidate. Atp6ap2 encodes the prorenin receptor and is functionally linked to V-ATPase biology. V-ATPase-dependent acidification is central to lysosomal function, autophagy, and protein turnover, processes that become increasingly important in aging and neurodegenerative disease [43,44]. The prorenin receptor and V-ATPase-mediated acidification also intersect with Wnt signaling in some contexts [45]. Loss of H3K27me3 at Atp6ap2 may therefore reflect an attempt to adjust lysosomal or proteostatic capacity in old neurons.

Heph, which encodes hephaestin, was also part of the broader loss module. Iron handling is relevant to brain aging because altered iron accumulation and metal homeostasis can contribute to oxidative stress and neurodegenerative vulnerability [46]. Heph was not included in the minimal 14-gene signature, but it is a strong candidate for an expanded stress and metal-homeostasis panel. Tspan7 and Msn add two additional themes. TSPAN7 regulates excitatory synapse development and AMPA receptor trafficking, suggesting possible synaptic membrane remodeling [47]. Moesin is part of the ERM family of cytoskeletal organizers, linking membrane structure to actin dynamics [48].

These loss events support the redistribution model. Aging neurons do not appear to undergo a simple, uniform decline in H3K27me3. Instead, the gain and loss modules point in different biological directions. Repression is gained at genes that maintain neuronal identity, synaptic adhesion, synaptic scaffolding, vesicle dynamics, and neuromodulatory responsiveness. At the same time, repression is lost at genes involved in transcriptional regulation, lysosomal biology, iron handling, synaptic membrane remodeling, and cytoskeletal coupling. This dual behavior could reflect compensatory adaptation, maladaptive stress activation, or a mixture of both.

Definition of the Synaptic Epigenetic Aging Signature

Integrating statistical strength, functional coherence, network position, and assay practicality yielded a compact 14-gene Synaptic Epigenetic Aging Signature (Table 9). The primary panel includes Pcdhb10, Pcdhb14, Pcdhgb4, Pcdhgc3, Nlgn3, Cask, Dlg4, Dlg3, Syn1, Chrm2, Chrna5, Chrnb4, Med14, and Atp6ap2. The first four genes capture protocadherin cluster repression and the proposed loss of neuronal barcode diversity. Nlgn3, Cask, Dlg4, Dlg3, and Syn1 capture synaptic adhesion, scaffolding, and vesicle regulation. Chrm2, Chrna5, and Chrnb4 capture cholinergic responsiveness. Med14 and Atp6ap2 capture the smaller loss-side biology of transcriptional rewiring and lysosomal adaptation.

**Table 9:** The 14-gene Synaptic Epigenetic Aging Signature, organized by functional module and predicted H3K27me3 direction in aged (24 M) neurons Dominant data-driven functional category: synaptic/neurotransmission genes. Expanded-panel candidates: Heph, Med12, Tspan7, and Msn. Recommended readout: H3K27me3 CUT&Tag-qPCR with matched isoform-aware RNA. CUT&Tag: cleavage under targets and tagmentation, Dir.: direction, H3K27me3: histone H3 lysine 27 trimethylation, qPCR: quantitative polymerase chain reaction, RNA: ribonucleic acid, V-ATPase: vacuolar-type H+-ATPase Gene symbols follow standard mouse gene nomenclature.

Module	Genes	Function	Dir.
Protocadherin identity	Pcdhb10, Pcdhb14, Pcdhgb4, Pcdhgc3	Neuronal “barcode” adhesion/self-avoidance	GAIN
Synaptic scaffold and vesicle	Nlgn3, Cask, Dlg4, Dlg3, Syn1	Adhesion-scaffold-vesicle release axis	GAIN
Cholinergic responsiveness	Chrm2, Chrna5, Chrnb4	Muscarinic/nicotinic receptors	GAIN
Transcriptional/lysosomal	Med14, Atp6ap2	Mediator complex; V-ATPase/proteostasis	LOSS

This panel is intended to be measured in two layers. The first layer is targeted H3K27me3 profiling, preferably by CUT&Tag-qPCR or another low-input chromatin method. The second is matched RNA expression, ideally with isoform-aware measurement for protocadherin genes. A practical validation experiment would test whether 24-month neurons show higher H3K27me3 at Pcdhb10, Pcdhb14, Pcdhgb4, Pcdhgc3, Nlgn3, Cask, Dlg4, Dlg3, Syn1, Chrm2, Chrna5, and Chrnb4, and lower H3K27me3 at Med14 and Atp6ap2. A stronger validation would then determine whether these chromatin states correlate with synaptic density, electrophysiological plasticity, and cognitive performance.

## Discussion

Mechanistic synthesis: Polycomb redistribution as a driver of circuit aging

**Figure 5:**
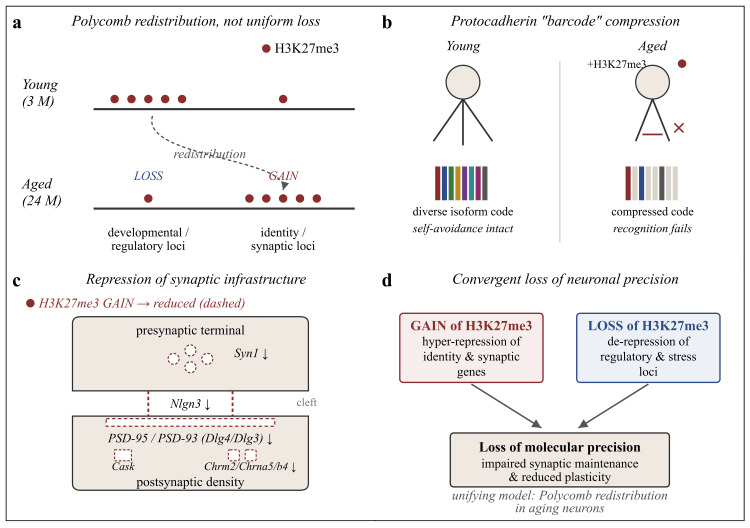
Proposed mechanism by which age-associated H3K27me3 redistribution may reduce neuronal precision (a) Rather than a uniform genome-wide decline, the aged 24-month neuronal Polycomb landscape is reorganized: repressive H3K27me3 is depleted at developmental and regulatory loci yet focally gained at neuronal-identity and synaptic loci. (b) Clustered protocadherins act as a combinatorial cell-surface “barcode” that enables single-neuron self/non-self recognition and dendritic self-avoidance. Focal H3K27me3 gain over the protocadherin clusters is predicted to compress this code, narrowing isoform diversity so that neighboring neurites may show impaired discrimination and weakened self-avoidance. (c) A coordinated adhesion-scaffold-vesicle-receptor module is simultaneously repressed: gain of H3K27me3 is predicted to dampen trans-synaptic adhesion through Nlgn3, postsynaptic scaffolds through PSD-95/PSD-93 and Cask, presynaptic vesicle mobilization through Syn1, and neuromodulatory receptors through Chrm2, Chrna5, and Chrnb4, weakening the molecular infrastructure that sustains mature synapses. (d) The opposing gain and loss arms are hypothesized to converge on a progressive loss of molecular precision, impaired synaptic maintenance, and reduced plasticity, providing a candidate epigenetic route from Polycomb redistribution to circuit-level vulnerability in the aging brain. All panels are schematic and depict hypotheses, not experimental measurements. Cask: calcium/calmodulin-dependent serine protein kinase, Chrm2: cholinergic receptor muscarinic 2, Chrna5: cholinergic receptor nicotinic alpha 5 subunit, Chrnb4: cholinergic receptor nicotinic beta 4 subunit, H3K27me3: histone H3 lysine 27 trimethylation, Nlgn3: neuroligin 3, Pcdh: protocadherin, PSD-95: postsynaptic density protein 95, PSD-93: postsynaptic density protein 93, Syn1: synapsin 1 Credits to Ngo Cheung Created using PowerPoint; no AI used

The central finding from this secondary analysis is that aging-associated H3K27me3 change can be organized into a coherent model of Polycomb redistribution (Figure 5). The source GSE190102 study showed broad loss of H3K27me3 in aged purified neurons, especially at developmental regulatory loci [25]. The present ABC-inspired analysis adds a complementary layer by identifying focal gains at genes required for neuronal identity and synaptic maintenance. Rather than viewing aged neurons as simply losing repression, this working model proposes that repression is misplaced: it is relaxed at some developmental, stress, or regulatory loci and intensified at selected connectivity genes.

This redistribution model helps connect chromatin biology to early circuit decline. Protocadherin gain suggests compression of neuronal molecular identity. Synaptic scaffold gain suggests weakened structural maintenance of synapses. Receptor gain suggests reduced neuromodulatory responsiveness. The combined effect is a neuron that may remain alive but become less distinct, less connected, and less adaptable. This is consistent with the idea that synaptic and circuit dysfunction can precede neuron loss and strongly influence cognitive impairment [2,3].

The protocadherin module is especially important because it links aging to neuronal individuality. During development, clustered protocadherins help establish self/non-self recognition, dendritic self-avoidance, and circuit assembly [10-12]. If aging represses selected members of these clusters, the effect may be a subtle erosion of the recognition system that keeps circuits precise. This is different from a generic synaptic decline model. It suggests that aged neurons may lose part of the molecular language that allows them to maintain their own wiring.

Translational implications: The Synaptic Epigenetic Aging Signature

The most immediate translational product of this work is not a drug but a measurable signature. The 14-gene Synaptic Epigenetic Aging Signature could serve as a candidate early biomarker of circuit vulnerability. A targeted H3K27me3 panel could be applied to aged mouse neurons, human induced neurons, organoids, or postmortem neuronal nuclei using low-input chromatin methods such as CUT&Tag [34]. If the signature appears before measurable synapse loss or behavioral decline, it could identify a window in which intervention is still likely to preserve function. This directly addresses the problem that many current biomarkers detect established molecular pathology, neurodegeneration, or downstream injury rather than the earliest molecular weakening of synaptic identity [1-7].

The same signature could also serve as a drug discovery endpoint. A major limitation of broad epigenetic therapy is the lack of specificity. Global PRC2 inhibition or nonspecific chromatin rejuvenation could destabilize cell identity, activate inappropriate developmental programs, or increase transcriptional noise, given the established role of Polycomb systems in developmental repression and cellular identity control [8,9,23,24]. The present results raise the possibility of a different goal: normalize H3K27me3 at selected synaptic identity loci without erasing necessary repression elsewhere. In screening systems, compounds or activity-dependent interventions could be ranked by whether they reduce H3K27me3 gain at Pcdhb10, Pcdhb14, Pcdhgb4, Pcdhgc3, Nlgn3, Cask, Dlg4, and Syn1 while preserving repression at inappropriate developmental genes.

The receptor component of the signature creates a route into treatment stratification. Cholinergic therapies and other neuromodulatory approaches show variable benefit in aging and neurodegenerative disease, and acetylcholine signaling has established roles in memory, attention, and cortical state regulation [21,22,40,41]. If high H3K27me3 at Chrm2, Chrna5, and Chrnb4 marks a state in which cholinergic receptor programs are epigenetically constrained, then this state may predict a weaker response to cholinergic enhancement. This is not yet established, but it is testable. A preclinical study could compare the chromatin state at cholinergic receptor genes with electrophysiological or behavioral response to cholinergic agents. A translational study could then ask whether analogous signatures in human neuronal models predict drug response.

The signature also has relevance for regenerative medicine. Transplanted, reprogrammed, or stem cell-derived neurons must integrate into host circuits, and that integration depends on molecular recognition, synaptic adhesion, and scaffold competence [10-20]. If the aged circuit has weakened protocadherin diversity and reduced synaptic scaffold competence, integration may fail not only because of inflammation or trophic support but because the molecular recognition and synaptic maintenance systems are compromised. Measuring and, eventually, restoring Pcdhb, Pcdhg, Nlgn3, Cask, and Dlg4 chromatin competence may improve integration strategies for neuronal replacement, retinal repair, spinal cord repair, and organoid transplantation models.

Finally, the signature suggests a disease-subtyping framework that could be called Synaptic Epigenetic Resilience. A protocadherin-repressed subtype would show high H3K27me3 at Pcdh clusters and may reflect early loss of circuit identity. A synaptic scaffold-repressed subtype would show high H3K27me3 at Nlgn3, Cask, Dlg4, Dlg3, and Syn1 and may reflect structural synaptic vulnerability. A neuromodulatory-repressed subtype would show high H3K27me3 at Chrm2, Chrna5, Chrnb4, and related receptor genes and may predict poor response to symptomatic neuromodulatory drugs. A stress-de-repressed subtype would show low H3K27me3 at genes such as Med14, Atp6ap2, and Heph and may reflect compensatory or maladaptive stress activation. Such subtypes could help explain why individuals with similar amyloid or tau burden often decline at different rates [5].

Comparison with existing literature and clinical relevance

The present model is compatible with several established lines of work. It fits the synapse loss literature by placing synaptic vulnerability upstream of overt degeneration [1,2]. It extends the cholinergic hypothesis by proposing that age-related cholinergic dysfunction may include an epigenetic receptor-competence component, not only loss of transmitter or projection neurons [21,22]. It also extends protocadherin biology beyond development. Protocadherins are best known for their roles in circuit assembly and self-avoidance, but long-lived neurons may continue to depend on these recognition systems for circuit maintenance [10,12].

The model also aligns with Polycomb biology. Polycomb complexes repress developmental regulators and stabilize cellular identity, but they are not static [9,23]. H3K27me3 can be redistributed in disease and environmental injury contexts, including memory impairment models [26]. In aging neurons, redistribution may have a double cost: loss of repression at developmental or stress loci may weaken identity boundaries, while gain at synaptic identity genes may reduce the molecular precision needed for circuit function.

Human translation will require careful model selection. Standard induced pluripotent stem cell-derived neurons may lose some age-associated signatures during reprogramming, whereas directly reprogrammed neurons can retain aspects of donor age [49,50]. For this reason, validation should include postmortem neuronal nuclei, directly induced neurons, and aged organoid or partial-aging systems when possible. Mouse forebrain findings should not be assumed to transfer directly to the human cortex or hippocampus, but they provide a tractable starting point.

Simulation-based reproducibility check of the differential-region pipeline

We first evaluated the differential-region workflow on a planted-signal dataset designed to mimic the small-sample structure of the neuronal H3K27me3 comparison, using standard false-discovery and differential-analysis frameworks [28,35-37]. The simulated dataset contained 4,000 genomic regions across eight samples, with four samples per condition. After median-of-ratios normalization and logCPM transformation, we compared limma empirical Bayes modeling [35], negative-binomial generalized linear modeling (NB-GLM) [36,37], and Welch testing. Limma and NB-GLM produced highly concordant differential-region calls, with a Jaccard index of 0.893. Against the planted truth, limma achieved a precision of 0.933 and a recall of 0.835, while NB-GLM achieved a precision of 0.887 and a recall of 0.867. Welch testing was more conservative, with high precision but substantially lower recall. These results supported the simulation benchmark as a robustness check for pipeline behavior under known-truth conditions; they did not replace the t-test-based primary biological analysis used for the reported GSE190102 region prioritization.

Within the simulated benchmark, threshold sensitivity analysis identified 148 regions recovered by all strategies and 595 regions recovered by at least one strategy. Using the simulated limma-eBayes criterion, 430 differential regions were identified, comprising 182 increased and 248 decreased simulated H3K27me3 regions. Of these simulated regions, 350 were assigned to at least one gene, yielding 632 simulated region-gene assignments and 598 unique genes. These simulation results were used to evaluate pipeline behavior under known-truth conditions, not as biological evidence for aging-associated H3K27me3 redistribution.

Limitations

Several limitations should shape interpretation. First, this is a secondary computational analysis of bulk purified neuronal data. Even though the source study purified NeuN-positive nuclei, the resulting signal still averages across neuronal subtypes. Single-nucleus CUT&Tag or joint chromatin-RNA profiling will be needed to determine whether the signature is broad across neurons or concentrated in vulnerable subtypes.

Second, the analysis remains limited by the small sample size of the source dataset. The relaxed discovery threshold and top-N fallback were chosen to preserve biological interpretability in a small secondary dataset, but they may increase false-positive findings. The differential regions should therefore be interpreted as statistically prioritized candidates requiring replication in independent cohorts.

Third, the ABC-inspired approach adapts a framework usually used for activating enhancer-gene assignment. Here, H3K27me3 signal was treated as repressive activity. Although the canonical ABC model was developed for active enhancers [29], the present adaptation treats H3K27me3 as a repressive proxy combined with estimated contact probability; this prioritizes candidate loci for hypothesis generation and does not replace direct chromatin conformation, promoter capture, or perturbation experiments.

Fourth, H3K27me3 gain does not automatically prove reduced expression. The relationship between this mark and transcription depends on local chromatin context, promoter state, enhancer architecture, and DNA methylation. For protocadherins, the most relevant expression change may be reduced isoform diversity rather than simple total expression loss. This makes isoform-aware RNA measurement essential.

Fifth, the analysis is based on mouse forebrain neurons and should not be assumed to translate directly to human cortical or hippocampal aging without validation in human neuronal systems.

Future directions

The next experiments should validate H3K27me3 gain at Pcdhb and Pcdhg loci, especially Pcdhb10, Pcdhb14, Pcdhgb4, and Pcdhgc3, and test whether this gain correlates with lower expression or reduced isoform diversity. Synaptic scaffold genes with H3K27me3 gain, including Nlgn3, Cask, Dlg4, Dlg3, and Syn1, should also be tested for reduced expression or reduced activity-dependent responsiveness in old neurons. Polycomb redistribution should then be examined directly by comparing broad H3K27me3 domain width, focal peak intensity, and PRC2 occupancy at EZH2- and SUZ12-bound regions.

A phased translational plan follows naturally. In the first phase, the 14-gene panel should be validated in independent aged mouse cohorts and human neuronal models using CUT&Tag-qPCR and matched RNA assays. In the second phase, causal testing should use epigenome editing or small-molecule screening in aged neurons or organoids. In the third phase, in vivo aged mouse studies and patient-derived neuronal stratification studies should test whether the signature predicts synaptic physiology, behavior, or drug response.

Therapeutic roadmap and ethical considerations

A future therapeutic strategy should not aim to erase H3K27me3 globally. The safer goal is locus-specific normalization. Programmable epigenome editing offers one possible route. dCas9-based systems can recruit chromatin regulators to selected loci, and related tools have already shown that transcriptional and epigenetic states can be manipulated in a targeted manner [51,52]. For the present model, a research-stage approach would be to recruit an H3K27me3 demethylating activity, such as KDM6A/UTX-related activity, to Pcdhb, Pcdhg, Nlgn3, or Dlg4 regulatory regions in aged neurons.

This remains far from clinical use. The risks are real. Protocadherin loci are complex, Polycomb repression protects cell identity, and inappropriate activation of developmental programs could be harmful. Any epigenome-editing strategy would need strong locus specificity, reversible control, long-term monitoring, and careful exclusion of germline applications. More realistic near-term interventions may include activity-dependent, neurotrophic, or small-molecule approaches that shift the signature toward a youthful pattern without directly editing chromatin. The signature can therefore be useful even before it becomes a therapeutic target: it can guide safer screening and reveal whether an intervention restores synaptic identity or merely produces broad chromatin disruption.

## Conclusions

This secondary analysis of publicly available neuronal H3K27me3 data supports a working model in which aging neurons undergo focal Polycomb redistribution. The key feature is not simply global loss of H3K27me3, although broad loss at developmental loci is an important part of the source dataset. The added insight is that aged neurons may also acquire focal H3K27me3 gain at genes required for neuronal identity, synaptic adhesion, postsynaptic scaffolding, vesicle dynamics, and neuromodulatory responsiveness. The strongest signal involves clustered protocadherins, especially the Pcdhb cluster together with Pcdhgb4 and Pcdhgc3. A second major signal involves Nlgn3, Cask, Dlg4, Dlg3, Syn1, Chrm2, Chrna5, and Chrnb4. A smaller loss-side signal involves Med14, Med12, Atp6ap2, Heph, Tspan7, and Msn. A proposed consequence, pending direct experimental validation, is a gradual loss of molecular precision. Aging neurons may become less able to maintain a diverse surface identity code, less able to stabilize mature synapses, and less responsive to neuromodulatory input. At the same time, selected loss of repression at transcriptional and stress-response loci may create compensatory or maladaptive flexibility. This dual dynamic provides a mechanistic bridge between chromatin aging and circuit decline.

The framework offers a way to approach five persistent difficulties in cognitive aging and neurodegeneration: late detection, blunt epigenetic therapies, variable drug response, poor regenerative integration, and clinical heterogeneity. The next step is experimental validation in independent mouse cohorts, single-nucleus neuronal datasets, human postmortem tissue, and age-preserving human neuronal models. If validated, focal H3K27me3 gain at protocadherin and synaptic maintenance loci may become a practical entry point for precision epigenetic approaches to cognitive aging.
